# Traditional Chinese medicine and its components effectively reduce resistance mediated by immune checkpoint inhibitors

**DOI:** 10.3389/fimmu.2024.1429483

**Published:** 2024-11-26

**Authors:** Mingxin Guo, Wentong Fang, Zhiqiang Hu

**Affiliations:** ^1^ Department of Pharmacy, The Affiliated Yixing Hospital of Jiangsu University, Yixing, China; ^2^ Department of pharmacy, Jiangsu Province Hospital, Nanjing, China

**Keywords:** tumor immunotherapy, immune checkpoint inhibitors, drug resistance, traditional Chinese medicine, Chinese herbs

## Abstract

Immunotherapy has become a global focus in cancer treatment and research, with promising results from targeting immune checkpoints in tumors like non-small cell lung cancer, colon cancer, and melanoma. However, resistance to immune checkpoint inhibitors (ICIs) remains a significant challenge. Traditional Chinese medicine (TCM), known for its low toxicity and minimal side effects, shows promise in enhancing cancer treatment when combined with modern therapies. This study reviews recent research on ICIs resistance mechanisms and highlights TCM’s potential in overcoming this resistance, aiming to improve ICIs efficacy while minimizing toxicity.

## 1 Introduction

Immune checkpoint inhibitors (ICIs) have revolutionized cancer treatment by harnessing the body’s immune system to target and eliminate tumor cells. They work by blocking immune checkpoints, such as programmed cell death-1 (PD-1), programmed cell death ligand 1 (PD-L1), and cytotoxic T-lymphocyte-associated protein 4 (CTLA-4), which tumors use to evade immune detection (1). By inhibiting these checkpoints, ICIs allow immune cells to recognize and attack cancer cells more effectively. This approach has led to significant advances in treating cancers like melanoma, non-small cell lung cancer, and renal cell carcinoma, offering durable responses in some patients who previously had limited treatment options (2, 3).

These ICIs are monoclonal antibodies that have high clinical effectiveness and specificity. However, they can also produce significant immunological resistance in hepatocellular carcinoma, microsatellite-stable colorectal cancer, hormone receptor-positive breast cancer, and other malignancies, which greatly impairs the efficacy of medications and prevents many patients from benefiting from them (4, 5). In recent years, the combination of ICIs with chemotherapy or targeted therapies has proven to be an effective strategy for overcoming immune resistance (6). At the same time, numerous studies have demonstrated that radiation can help treat drug-resistant cancers by altering the tumor microenvironment (TME) (7). However, the use of combination treatment significantly raises the risk of severe immune-related adverse events (IRAEs) in patients (8).

Traditional Chinese Medicine (TCM) provides an interesting perspective on tumor biology, including *yin* and *yang*, *qi* and blood circulation issues, and organ dysfunction. TCM experts have steadily realized the importance of strengthening immunity in tumor treatment. As a result, the therapeutic concept of balancing *yin* and *yang* is frequently related to immune function and immune balance. As a result, by leveraging the gentle nature and low side effects of TCM, we can potentially intervene in drug resistance associated with ICIs. This article explores the mechanisms underlying ICIs resistance and investigates the potential role of TCM in mitigating this resistance, offering fresh perspectives for future research. Our goal is to combine TCM with Western medicine to overcome ICIs resistance and optimize outcomes in tumor immunotherapy.

## 2 Intrinsic factors of resistance mediated by ICIs

The mechanism of action for ICIs continues to advance. Broadly, ICIs work by attenuating the inhibitory effects of negative co-stimulatory molecules like CTLA-4 and PD-1/PD-L1 on T cell. This prevents the deactivation of activated T cell and the evasion of tumor cells, enabling T cell to effectively destroy tumor cells without restraint (9, 10). The process of tumor killing by ICIs is complex and influenced by various factors. Consequently, their resistance mechanisms are equally diverse and intricate, as illustrated in **Table 1**. Drug resistance can be classified into three categories based on the timing of its occurrence. Primary drug resistance happens when the tumor shows no initial response to ICIs therapy. Acquired drug resistance occurs when an initial successful response to ICI therapy is followed by disease progression or relapse after a period of treatment. Adaptive drug resistance refers to the ability of tumor cells to evade immune-mediated killing (11, 12).

**Table 1:** Mechanisms associated with tumor immune resistance.

Factor	Type	Mechanism
Intrinsic factors	Genetic Changes	1. The lossing of gene copy number 2. Clonal selection and epigenetics are suppressed 3. Weakening of microsatellite instability 4. The genes and transcription of MHC-I were abnormal 5. The absence of *PTEN*
	Signaling Pathway Alterations	1. IFN-*γ* signaling pathway 2. WNT/*β*-catenin signaling pathway 3. Abnormal function of DC 4. Reduction of tumor mutational burden
Extrinsic factors	TME Changes	1. Inhibition of T cell transport chemokine expression 2. Blocking T cell infiltration 3. Massive proliferation of immunosuppressive cells 4. Altering the supply and metabolism of substances in T cell 5. Lack of TIL in TME
	Others	1. Upregulation of other inhibitory immune checkpoints(IDO1、CD73、A2AR) 2. Effect of gut flora *in vivo*

### 2.1 The changes in tumor cells themselves

The identification of tumor antigens by effector T cell is particularly crucial in immunotherapy, as changes in tumor antigens or their abundance can influence the recognition and elimination of these cells (13). A higher tumor mutational burden (TMB) has been associated with improved overall survival following immunotherapy in certain cancer types. Current studies indicate that malignant melanoma, renal cell carcinoma, and non-small cell lung cancer (NSCLC) generally exhibit higher TMB compared to pancreatic and prostate cancers. Consequently, the former group tends to show higher sensitivity to PD-1/PD-L1 treatment, whereas the latter group exhibits lower sensitivity (14, 15). Moreover, in certain malignancies, an increase in DNA mismatch repair (dMMR) gene deletions and high genomic microsatellite instability (MSI-H) can result in elevated levels of tumor-associated neoantigens. These neoantigens can potentially enhance the efficacy of ICIs against treatment resistance. Conversely, when the tumor’s mutational burden decreases and the dMMR/MSI-H ratio is low, there may be reduced production of tumor-associated antigens, contributing to resistance to treatment (16, 17).

### 2.2 The changes in signaling pathways

Signaling mechanisms regulating endogenous tumor antigens can modify them, impeding T cell detection. Activation of the mitogen-activated protein kinase (MAPK) signaling pathway induces the production of vascular endothelial growth factor and interleukin-8 (IL-8). These factors can diminish T cell recruitment and impair effector T cell (Teff) infiltration in malignancies (18). The deletion of the tumor suppressor gene *PTEN* is more common in malignant cancers. Loss of *PTEN* enhances the activity of the phosphatidylinositol-3-kinase/protein kinase (PI3K/AKT) signaling pathway, which in turn reduces the expression levels of interferon-γ (IFN-*γ*) and granzyme B genes. This decrease ultimately hampers the infiltration of CD8+ T cell into tumors (19). In patients with malignant melanoma, *PTEN* deletion is associated with reduced tumor site T cell infiltration, a lower chance of T cell growth following tumor excision, and inferior PD-1 inhibitor therapy (20).

INF-*γ* contributes to primary, adaptive, and acquired drug resistance and has a bidirectional influence on tumor immune response (17). Tumor-specific T cell produce IFN-*γ*, which recognizes tumor cells and their specific antigens. This enhances the expression of protein molecules such as major histocompatibility complex (MHC), facilitating antigen presentation, immune cell recruitment, and effector molecules that either inhibit tumor proliferation or promote apoptosis (21). Tumor cells lacking the IFN-*γ* signaling pathway are less vulnerable to T cell attacks, leading to resistance against ICIs. Prolonged exposure to IFN-*γ* can induce tumor cells to undergo redifferentiation, evading detection by the immune system. Mutations or deletions in receptor chains such as janus kinase (JAK), signal transducers and activators of transcription (STAT), and interferon regulatory factor 1, which are crucial in the IFN-*γ* signaling cascade, contribute to immune evasion by tumor cells (22).

The WNT/*β*-catenin signaling pathway impacts T cell recruitment and promotes non-T cell infiltration into the TME by inhibiting chemokine C-C ligand 4 and dendritic cell (DC) aggregation. Additionally, it regulates the TME through interactions with tumor-associated macrophages, promoting lactate production, and inhibiting the production of cytotoxic T lymphocytes. Consequently, this pathway diminishes the therapeutic efficacy of PD-1/PD-L1 inhibitors (23). The activation of the WNT signaling pathway promotes the expression of *β*-catenin, potentially acting as an oncogene. In mouse models, tumors expressing high levels of *β*-catenin show a decreased presence of CD103+ dendritic cell (DC) within the TME. This reduction is attributed to lower levels of the chemokine *CCL4*, which impairs antigen presentation. Consequently, this alteration affects T cell infiltration and compromises the immune response in the microenvironment (24).

### 2.3 The expression of constitutive PD-L1

Tumor cells can produce immunosuppressive ligands on their surfaces, such as PD-L1, which effectively inhibit anti-tumor T cell responses. The PDJ amplicon, found on chromosome 9, comprises the PD-L1, PD-L2 and INF-*γ* receptor signal transduction molecule JAK2. Therefore, the expression of PDJ may lead to an increase in the expression of PD-L1 (25). Studies have discovered that the mechanism of tumor-acquired drug resistance may be related to mutations in the IFN-*γ* signaling pathway, changes in target antigens, and secondary changes in the molecular structure of human leukocyte antigen 1, which may be related to the loss of antigen expression or antigen presentation function. Defects are connected, resulting in T cell’ inability to identify tumor cells (26). During treatment with ICIs, initial mutations can disappear, leaving behind a more complex new mutation and T cell profile that no longer depends on the PD-1 pathway, resulting in resistance to ICIs. Mutations in *β*-2 microglobulin of tumor surface antigens may lead to the loss of human leukocyte antigen, impairing the effective presentation of tumor antigens on the surface of tumor cells and thereby reducing the recognition function of CD8+ T cell (27).

## 3 Extrinsic factors of resistance mediated by ICIs

The main external factors causing drug resistance are tumor immune microenvironment components, such as myelosuppressive cell (MDSC), regulatory T cell (Treg), M2 macrophages, and immunosuppressive substances, which may inhibit the anti-tumor immune response and thus affect the anti-tumor effect of ICIs (28).

### 3.1 Tumor immune microenvironment components

Tumor cells often aggregate with a high number of immune cells and their microenvironment, forming a protective barrier against malignancies. However, once breached, this barrier can significantly influence tumor progression. MDSC constitute a diverse myeloid cell population crucial in the TME, particularly during malignancy and chronic infections. MDSC play pivotal roles in neovascularization, tumor cell invasion, and metastasis, thereby facilitating tumor growth. They profoundly suppress the antitumor activities of T cell, natural killer (NK) cell, and certain myeloid cells, while promoting the expansion of Treg (29, 30). Treg play a crucial role in maintaining self-tolerance. They can release inhibitory cytokines such as IL-10, IL-35, and transforming growth factor-beta (TGF-*β*), or directly suppress the response of Teff (31). Experimental studies in mouse models have demonstrated that reducing Treg in the TME effectively mitigates tumor immune resistance. Additionally, research has shown that the therapeutic efficacy of a CTLA-4 inhibitor is associated with the ratio of Teff to Treg; higher ratios correlate with better therapeutic outcomes (32, 33). Tumor-associated macrophages (TAM) are another critical cell type influencing the efficacy of immunotherapy. TAM encompasses both M1 and M2 macrophages. M1-type macrophages enhance antitumor immunity, whereas M2-type macrophages tend to promote tumor growth (34, 35).

### 3.2 Immunosuppressive substance

The immunological response in a healthy human body is finely tuned and balanced. It stimulates the anti-pathogen response while also controlling the activation of suppressor genes and pathways to prevent overreaction that could harm the body itself. The T cell receptor signaling pathway and CD28 play crucial roles in boosting T cell activation, which ultimately leads to increased expression of CTLA-4 (36). Tumors and macrophages often secrete immunosuppressive substances that hinder immune responses against tumors. During the Teff response, increased production of IFN-*γ* promotes the expression of PD-L1 on the surfaces of various cells, including tumor cells, T cell, and macrophages. This results in heightened binding of PD-L1 to PD-1 on T cell surfaces, thereby enhancing immunosuppressive effects (37).

IDO1, an enzyme that converts tryptophan to kynurenine, is also an immunosuppressive molecule that specifically drives tumor immunosuppression, neovascularization, and metastasis (38). IDO1 may decrease the function of local CD8+ T cell and NK cell, stimulate the generation of CD4+ Treg, control the recruitment of MDSC, and inhibit their function (39). The movement of MDSC and Treg in the TME depends on specific chemokines and chemokine receptors. Ligands such as *CCL5*, *CCL7*, and *CXCL8* released by tumor cells bind to receptors *CCR1* and *CXCR2* expressed on MDSC, causing them to cluster and migrate chemotactically towards the TME (40). CD73 is an ectoenzyme derived from adenosine that catalyzes adenosine monophosphate to adenosine. It inhibits immune function, promotes tumor cell dissemination, and stimulates angiogenesis. CD73 is also considered a potential biomarker for PD-1 therapy, with high levels of expression associated with poor outcomes (41). High levels of TGF-*β* and CD73 have been linked to poor prognosis in several cancers (42). Adenosine suppresses T cell proliferation and cytotoxicity through the adenosine 2A receptor on T cell surfaces, while promoting tumor metastasis via the adenosine 2B receptor on tumor cells.

### 3.3 Other factors

The absence of tumor-infiltrating lymphocytes (TIL) within the TME is a major factor contributing to resistance to ICIs. Persistent anti-tumor activity can lead to T cell depletion. Consequently, cancers that lack T cell both inside the tumor and in its microenvironment are categorized as “cold tumors”, characterized by a “desert type” microenvironment. Severe T cell depletion may eventually lead to secondary immune resistance (43). Studies have shown that ICIs alone may not effectively target cold tumors, which lack significant immune cell infiltration. Combining ICIs with agents that enhance immune cell activation and infiltration can improve their ability to suppress tumors. This highlights the importance of combining immune stimulants with ICIs for treating cold tumors (44). However, there has been limited research on the specific processes underlying this relationship. Only a few researchers have suggested a potential connection to mechanisms involving inflammatory cell death (45). The gut flora can be considered as the human body’s second genome, and imbalances in gut flora may potentially compromise the effectiveness of ICIs therapy. However, the specific interaction mechanisms between gut flora and ICIs treatment remain unknown (46, 47).

## 4 The difference of the concept of western and traditional medicine

In Western medicine, the approach to cancer treatment is often centered on identifying and targeting specific molecules or pathways associated with tumor growth and metastasis. This has led to the development of therapies such as targeted therapies (e.g., monoclonal antibodies), small molecule inhibitors, and immunotherapies (e.g., ICIs) that aim to directly interfere with cancer cells or their environment (48). This approach is very focused on the specificity of the treatment and its ability to selectively target tumor cells without causing harm to healthy cells.

In contrast, TCM employs a more holistic view of disease and treatment, often emphasizing the balance of the body’s energy (*qi*), as well as the regulation of the internal environment through complex mixtures of herbs and other natural substances. These treatments typically involve combinations of multiple compounds, each of which may target different aspects of the body’s functioning. In the context of cancer, TCM often aims not just at directly killing cancer cells but at strengthening the body’s immune system, improving *qi* circulation, balancing *yin* and *yang*, and restoring harmony to the body’s various systems (49). The efficacy of TCM for cancer treatment is often not solely dependent on killing cancer cells directly, but rather on the regulation and enhancement of the body’s immune response, which may help the body fight cancer in a more systemic, rather than targeted, way.

The concept of “immune regulation” is central to many cancer therapies in TCM. TCM practitioners often emphasize strengthening the body’s “*Zheng qi*” to fight cancer. By enhancing the immune system’s ability to detect and destroy cancer cells, TCM may help enhance the body’s natural defenses (50). There are many herbs used in TCM that are believed to enhance immune function, such as Huang qi, Ling zhi, and Ren shen, all of which have been studied for their potential to modulate immune responses, stimulate white blood cell activity, and balance inflammation. Moreover, some studies have suggested that TCM may complement or synergize with conventional cancer therapies. For example, certain TCM herbs might reduce the side effects of chemotherapy, enhance its effectiveness, and improve overall patient quality of life by supporting immune health and reducing fatigue or nausea (51).

## 5 TCM mechanisms and efficacy

When it comes to the immune system, TCM often categorizes treatments based on whether they need to boost or regulate the immune response, considering the patient’s overall condition and specific imbalances. To further demonstrate this, **Table 2** categorizes several typical TCM ways for activating or regulating the immune system based on various underlying patterns. *Qi* refers to the vital energy of the body, which must be abundant for immunity and overall health. When *qi* is deficient, the body’s defenses become weak. *Yin* represents cooling, moistening, and nourishing aspects of the body, while *Yang* represents warmth, activity, and energy. In TCM, both need to be in balance for optimal health. If the immune system is overactive, it can lead to *Yang* excess, whereas insufficient *Yang* can lead to a weak immune system. Blood in TCM represents the nourishment and cooling aspects of the body. Blood deficiency can lead to poor circulation and low energy, weakening the immune system.

**Table 2:** Categorized TCM approaches for immune system regulation.

Category	Treatment Focus	Herbal examples	Conditions Treated
Strengthening the Immune System (*Qi* Deficiency)	Activating the immune system by tonifying *Qi*	*Ginseng Radix et Rhizoma* (Ren shen)*, Astragali Radix* (Huang qi)*, Codonopsis Radix* (Dang shen)*, Atractylodis Macrocephalae Rhizoma* (Bai zhu)	Chronic fatigue, weak immunity, recurrent colds, general weakness, post-illness recovery
Tonifying the Immune System (Blood Deficiency)	Strengthening both *Qi* and Blood to boost immunity	*Angelicae Sinensis Radix* (Dang gui)*, Rehmanniae Radix Praeparata* (Shu dihuang)*, Paeoniae Radix Alba* (Bai shao)*, Chuanxiong Rhizoma* (Chuang xiong)	Anemia, fatigue, skin conditions, slow recovery from illness, menstrual issues, general weakness and lethargy
Regulating the Immune System (Wind-Heat or Wind-Cold)	Resolving pathogenic factors such as Wind-Heat or Wind-Cold	*Menthae Haplocalycis Herba* (Bo he)*, Lonicerae Japonicae Flos* (Jin yinhua)*, Glycyrrhizae Radix et Rhizoma* (Gan cao)*, Bupleuri Radix* (Chai hu)	Common cold, flu, respiratory infections, seasonal illnesses, allergic reactions like rhinitis and asthma
Calming the Immune Response (*Yin* Deficiency with Heat)	Nourishing *Yin*, cooling the body, and regulating the immune system	*Lilii Bulbus* (Bai he)*, Rehmanniae Radix* (Shu dihuang)*, Ophiopogonis Radix* (Mai dong)*, Scrophulariae Radix* (Xuan shen)	Autoimmune conditions, excessive inflammation, fever, hot flashes, skin conditions like eczema or psoriasis
Anti-inflammatory Immune Modulation	Modulating inflammation while strengthening immune regulation	*Yu ping feng san, Shen ling bai zhu san*	Chronic inflammation, allergies, autoimmune conditions, inflammatory bowel diseases like Crohn’s disease
Calming the Nervous System (Excessive *Yang*/*Heat*)	Rebalancing the nervous system, cooling excessive *Yang* or heat	*Ostreae Concha* (Mu li)*, Cinnamomi Ramulus* (Gui zhi)*, Lilii Bulbus*	Stress-induced immune dysregulation, anxiety, insomnia, skin problems caused by heat or stress
Supporting the Immune System in Cold/Weak Constitution	Boosting Yang to support overall vitality and immune defense	*Cinnamomi Cortex* (Rou gui)*, Aconiti Lateralis Radix Praeparata* (Fu zi)*, Cervi Cornu Pantotrichum* (Lu rong)	Chronic cold hands and feet, low energy, weak digestion, frequent illnesses due to weak immune response

## 6 Study on drug resistance of immunotherapy controlled by TC

### 6.1 Study into the regulation of PD-1/PD-L1 by TCM and its active components

Chinese herbs have been shown to modulate the immunosuppressive TME, enhance CD8+ T cell infiltration, and inhibit tumor growth as illustrated in **Table 3** (52). Researchers have demonstrated in studies involving Lewis lung cancer mice and humanized PD-1 knockout mice that ginseng polysaccharides, when injected with PD-1 inhibitors, can alter the gut microbiota and tryptophan ratio. This alteration enhances the anti-PD-1/PD-L1 immunotherapy’s effectiveness (53). Furthermore, ginseng-derived nanoparticles have been found to reprogram TAM, leading to increased release of *CCL5* and *CXCL9*. This reprogramming synergizes with PD-1 treatment by recruiting CD8+ T cell into the tumor (54).

**Table 3:** Study into the regulation of PD-1/PD-L1 by TCM and its active components.

Substance	Mechanism
Ginseng polysugar	Regulation of intestinal flora and recruitment of CD8+ T cell
Astragalus polysaccharide	Regulation lipid metabolism; Increase the number of CD4+ T cell; Reduce PD-L1 expression and protein levels
Artesunate	Mediating immune resistance in tumors by modulating PD-L1; Recruitment of infiltrating CD8 + T cell to overcome the development of ICIs mediated resistance; Promoting cancer cell death by inducing ferroptosis
Andrographolide	Regulating the production of tumor tissue growth factor; increasing tumor suppressor cytokines; inducing death of tumor cells
Atractylodis macrocephalae rhizoma polysaccharide	Promoting miR-34a overexpression while decreasing PD-L1 expression; inhibiting the activation of the NRF2 pathway. Regulation of the *PTEN*/PI3K/AKT signaling pathway reduces tumor development
Cryptotanshinone	Increasing infiltration of CD8 + T and CD4 + T cell in TME; inhibiting cancer cell cycle
Quercetin	Reducing PD-L1 inhibitory impact on T cell and increase expression of granzyme B and IFN-*γ*
Curcumin	Promoting tumor antigen-specific T cell induction *in vivo*; inhibiting immune evasion by reducing transforming growth factor-*β*1 expression
*β*-elemene	Inducing ferroptosis in cancer cells; regulating AMPK/MAPK, JAK2/STAT3, PI3K/AKT, and other signaling pathways to reverse multidrug resistance
Galangin	Inhibition of PD-L1 expression on tumor surface; inhibition of neovascularization of tumor vasculature
Rocaglamide	Promoting NK cell invasion; Promoting the proliferation of tumor-infiltrating T cell

Astragalus polysaccharide (APS) treatment significantly increased the ratio of CD4+ T cell and the CD4/CD8 T cell ratio in mice with lung cancer, along with elevating blood levels of IFN-γ and IL-2. APS also reduced the expression of PD-L1 in tumor tissue (55). APS is known to activate signaling pathways such as IL-6/STAT3 and nuclear factor kappa-B (NF-κB), among others. This activation promotes the differentiation of bone marrow mesenchymal stem cells into tumor-associated fibroblasts and regulates lipid metabolism (56). Studies by Chang et al. have demonstrated that APS can reduce the expression and protein levels of PD-L1 in tumor cells, both *in vivo* and *in vitro*. This action suggests APS’s potential to overcome immune escape and resistance to immune therapy (57).

Artesunate, an artemisinin derivative derived from *Artemisiae Annuae Herba*, exhibits potent antitumor efficacy with minimal toxicity (58). The PDZ-binding motif TAZ mediates immunological resistance in tumor cells by regulating PD-L1. Initially, research identified that artesunate targets TAZ-TEAD complexes by binding a hydrophobic group securely to the complex’s hydrophobic pocket, thereby promoting TAZ degradation through a proteasome-dependent pathway. Artesunate also reverses ICIs mediated resistance in NSCLC by facilitating infiltration of CD8+ T cell (59). Artesunate also promotes ferroptosis by downregulating glutathione peroxidase 4 and reduces T cell growth inhibition mediated by TAZ/PD-L1, leading to cancer cell death (59). However, further research is needed to fully elucidate its mechanism of action and safety profile. Artesunate is anticipated to emerge as a promising novel anti-tumor therapy for clinical applications in the near future.

Andrographolide, the main active compound in *Andrographis Herba*, regulates tumor tissue growth factors, suppresses tumor development, induces tumor cell death, and enhances infiltration and activity of CD4+ and CD8+ T lymphocytes (60). Furthermore, andrographolide enhances tumor-suppressing cytokines including TNF-*α*, IFN-*γ*, perforin, and glutamate B. Liu et al. demonstrated in a CT26 colon cancer mouse xenograft model that combining andrographolide with an anti-PD-1 antibody achieves superior therapeutic efficacy compared to monotherapy. This enhanced effect may involve inhibition of cyclooxygenase-2 activity, reduction in prostaglandin E2 production, and improved PD-1 functionality (61).

Atractylodis macrocephalae rhizoma polysaccharide (AMRP) has been demonstrated to exert beneficial pro-immune effects. miRNA sequencing has shown that Atractylodis macrocephala polysaccharide may alleviate immunosuppression by upregulating the TCR-NFAT pathway through novel-mir2 targeting CTLA-4 (62). Additionally, miR-34a targets the PD-L1 gene. Overexpression of miR-34a following transfection for 48 hours suppressed PD-L1 mRNA expression, while Atractylodes macrocephala polysaccharide increased miR-34a expression and reduced PD-L1 levels. This suggests AMRP could potentially serve as a significant anti-tumor agent, capable of mitigating resistance mediated by ICIs (63).

Research has shown that reduced expression of the nuclear factor erythroid 2-related factor 2 (NRF2) signaling pathway can significantly increase infiltration of CD8+ and CD4+ T cell into tumors, inhibit melanoma growth, and decrease PD-L1 expression. Atractylodis macrocephalae rhizoma polysaccharide (AMRP) is known to enhance NRF2 ubiquitination, leading to its degradation and subsequent inhibition of NRF2 pathway activity (64). AMRP functions as an NRF2 inhibitor, potentially reducing tumor progression through modulation of the *PTEN*/PI3K/AKT signaling pathway and inhibition of proto-oncogenes, thereby promoting degradation of hypoxia-inducible factor-1α. Researchers have suggested that combining AMRP with ICIs could enhance anti-tumor effects while improving immune tolerance (65, 66).

Cryptotanshinone has been found to enhance the infiltration of CD8+ and CD4+ T cell in the TME and promote the high production of *CXCL9*, *CXCL11*, and granzyme B. Its impact is further enhanced when combined with anti-PD-L1 therapy. In a Lewis lung cancer mouse model, Liu et al. discovered that cryptotanshinone reduced tumor cell growth by increasing P53 expression and decreasing cyclin B1 and Cdc2 levels. Additionally, salvianolic acid B has shown potential to increase CD8+ T cell infiltration in the TME while preserving vascular endothelial integrity, thereby restoring normal vascular function. It also enhances the effectiveness of anti-PD-L1 therapy in breast cancer models (67, 68).

Quercetin, a flavonoid commonly found in *Curcumae Longae Rhizoma*, *Platycodonis Radix*, and *Psoraleae Fructus*, exhibits the ability to overcome drug resistance in various cell types and modulate human immune function through interactions with the HCV, HSV-1, H1N1, HBV, and HIV-1 signaling pathways. Studies have demonstrated that quercetin reduces PD-L1-mediated suppression of T cell by blocking the PD-1/PD-L1 interaction. Additionally, quercetin enhances the production of granzyme B and IFN-*γ* (69, 70).

Curcumin, the primary active compound in *Curcumae Longae Rhizoma*, a TCM known for its immunomodulatory properties, has shown significant effects in two animal tumor models (MC38 and CT26). Curcumin enhances the induction of tumor antigen-specific T lymphocytes *in vivo* by restoring dendritic cell function. This is achieved through the suppression of STAT3 signaling in dendritic cells, which indirectly reduces IL-6 production by tumor cells (71). Curcumin, in combination with PD-1 inhibitors, synergistically reduces Hep3B cell proliferation, prevents immune evasion, and lowers TGF-*β*1 expression (72). Moreover, the combination of curcumin with PD-1/PD-L1 inhibitors demonstrates potent antitumor effects, especially in cancer patients exhibiting high IL-6 expression (71).

*β*-elemene, an active component derived from *Curcuma Longae Rhizoma*, suppresses tumor growth and controls the immune system (73). Chen et al. recently revealed that *β*-elemene induces ferroptosis. When combined with cetuximab, it can induce ferroptosis, increasing the sensitivity of colorectal cancer cells with KRAS mutations. It can also promote ferroptosis in cancer cells via the pole2-mediated *P53* and PI3K/AKT signaling pathways (74). Recent investigations have indicated that *β*-elemene can enhance the expression of *PTEN* by METTL3-mediated N6 methyl adenosine modification. However, the particular mechanism of action has not been thoroughly studied. Furthermore, *β*-elemene can reverse multidrug resistance by regulating signaling pathways such as AMPK/MAPK, JAK2/STAT3, and PI3K/AKT (75).

Galangin is an antiproliferative, anti-inflammatory, pro-apoptotic, anti-angiogenic, and metastatic chemical derived from *Alpiniae Officinarum Rhizoma* that has strong anticancer activity. Galangin may also enhance the expression of *PTEN (* 76). Galangin inhibits vascular injury-induced neointimal formation through the suppression of MAPK and NF-*κ*B signaling pathways. Conversely, NF-*κ*B ligand activators promote osteoclast formation by stimulating NF-*κ*B signaling pathway (77). Galangin has been demonstrated to inhibit the JAK/SRC and RAS/RAF/MEK/ERK signaling pathways, thereby reducing the activation of STAT3 and Myc, respectively. Furthermore, inhibiting the interaction between STAT3 and Myc decreases the expression of the PD-L1 protein on tumor surfaces. This inhibition reduces the binding of PD-L1 on tumor cells to PD-1 on T cell, consequently restoring T lymphocyte viability and enhancing tumor cell-specific lethality (78).

In melanoma models, Yu et al. reported that alitunone enhances the effectiveness of anti-PD-L1 therapy by downregulating C-JUN expression and reducing Treg activity through the C-JUN-PD-L1 pathway, thereby delaying disease progression (79). Rocaglamide, a TCM monomer derived from the *Morella Rubra* Lour., increases NK cell infiltration and boosts anti-tumor immunity by inhibiting autophagy and activating the cGAS-STING signaling pathway. Rocaglamide has the capacity to convert cold tumors into hot tumors, thereby promoting the proliferation of tumor-infiltrating T cell (80).

### 6.2 Study on the regulation of PD-1/PD-L1 by TCM prescriptions

As illustrated in **Table 4**, Gegen Qinlian decoction (GQD) has a positive therapeutic impact on colorectal cancer. Immune checkpoint drugs have a modest therapeutic impact on microsatellite-stabilized colorectal cancer, and the immune system is extremely resistant. GQD regulates the lipid metabolic route of sphingomyelin and glycerophospholipid, improves the anti-tumor effectiveness of ICIs, inhibits tumor cell immune escape, and works with PD-1 inhibitors by modulating the Wnt/*β*-catenin signaling pathway. Regulate the gut flora and change the TME (81). Shenqi Fuzheng Injection (SFI) is a clinically utilized adjuvant therapy for individuals with lung and stomach cancer. SFI enhances the immunological milieu of melanoma tumors by lowering MDSC and Treg and enhancing CD8+ T cell and CD4+ T cell through fatty acid metabolism. Finally, this enhancement leads to greater antitumor effects of the immune checkpoint inhibitor PD-L1 antibody (82). Qingfei Jidu decoction (QJD) reduced EGFR, JUN, RELA, HIF1A, NFKBIA, and CD274 expressions while increasing AKT1 and MAPK1 levels in A549 cell. Lewis lung carcinoma tissue in the QJD high dosage group had a lower PD-L1 IRS than the model group. CD8+PD-1+T% was greater in the QJD high dosage group compared to the normal and model groups (83).

**Table 4:** Mechanisms by which TCM Prescriptions regulate PD-1/PD-L1.

TCM Prescriptions	Composition	Mechanism
Gegen Qinlian decoction(GQD)	*Puerariae Lobatae Radix* (Ge gen)*、Scutellariae Radix* (Huang qin)*、Coptidis Rhizoma* (Huang lian)*、Glycyrrhizae Radix et Rhizoma*	Regulating Wnt/*β*-catenin signaling pathway and lipid metabolism; Preventing tumor cells from escaping the immune system. Coordinating with PD-1 inhibitors to balance the intestinal flora and change the TME.
Shenqi Fuzheng Injection (SFI)	*Codonopsis Radix、Astragali Radix*	Regulating tumor fatty acids; Reducing MDSC and Treg; Increasing CD8+ T cell and CD4 + T cell.
Qingfei Jidu decoction(QJD)	*Scutellariae Radix, Citri Reticulatae Pericarpium* (Chen pi)*, Ophiopogonis Radix, Fritillariae Thunbergii Bulbus* (Zhe beimu)*, Paeoniae Radix Rubra* (Chi shao)*, Mori Cortex* (Sang baipi)*, Glycyrrhizae Radix Et Rhizoma, Coptidis Rhizoma, Taraxaci Herba* (Pu gongying)	Regulating of the HIF-1, EGFR, JUN, and NF-*κ*B signaling pathways.
Jianpi Zishen Xiehuo formula (JZXF)	*Astragali Radix, Ligustri lucidi Fructus* (Nv zhenzi)*, Cuscutae Semen* (Tu sizi)*, Rehmanniae Radix, Moutan Cortex* (Mu danpi)*, Atractylodis Macrocephalae Rhizoma, Paeoniae Radix Alba, Rubiae Radix Et Rhizoma* (Qian cao)*, Agrimoniae Herba* (Xian hecao)*, Selaginellae Herba* (Juan bai)*, Rhodiolae Crenulatae Radix Et Rhizoma* (Hong jingtian)*, Notoginseng Radix Et Rhizoma* (San qi)*, Aurantii Fructus* (Zhi qiao)*, Glycyrrhizae Radix Et Rhizoma*	Influence the expression of complement regulating proteins CD55 and CD59.
Buzhong Yiqi decoction (BYD)	*Astragali Radix、Atractylodis Macrocephalae Rhizoma、Citri Reticulatae Pericarpium、Cimicifugae Rhizoma* (Sheng ma)*、Bupleuri Radix、Ginseng Radix Et Rhizoma、Glycyrrhizae Radix Et Rhizoma、Angelicae Sinensis Radix*	Regulating TME, increasing IFN-*γ* levels, and reducing PI3K/AKT signaling pathway activity
Yangyin Fuzheng Jiedu prescription (YFJP)	*Astragali Radix、Atractylodes macrocephala Koidz、Radix Bupleuri、Hedyotis diffusae Herba* (Baihua sheshecao)、*Radix Sophorae flavescentis、Radix Cynanchi paniculati、Glehniae Radix* (Bei shashen)*、Ophiopogon japonicus*	Relieving T cell depletion; regulating TME; regulating cytokine release
Dahuang Fuzi Baijiang decoction (DFBD)	*Radix et Rhizoma Rhei* (Da huang)*、Radix Aconiti Lateralis* (Fu zi)*、Praeparata、* *Herba Asari* (Xi xin)*、Herba Patriniae* (Bai jiangcao)*、Semen Coicis* (Yi yiren)	Relieving T cell depletion; Regulating TME

Jianpi Zishen Xiehuo Formula (JZXF) is commonly used to treat primary immune thrombocytopenia. Li et al. discovered that the mRNA and protein expression of JAK1 and STAT1 was dramatically reduced in the drug-containing serogroup (20 g/kg) using western blot and CCK-8 (84). It also has a therapeutic impact on ITP by suppressing the JAK/STAT signaling system. Clinical investigations have shown that it can alter the expression of complement regulatory proteins CD55 and CD59, which are crucial strategies to increase resistance to ICIs (85). Buzhong Yiqi decoction (BYD) as an adjuvant treatment for gastric cancer patients following chemotherapy has the potential to considerably increase patient survival time. Further investigation revealed that BYD decreased PD-L1 expression in gastric cancer through the PI3K/AKT pathway. BYD has the potential to modify peripheral immunity and prevent tumor immune escape, making it a viable treatment for gastric cancer (86). Yangyin Fuzheng Jiedu prescription (YFJP) is a traditional Chinese herb decoction that has long been used in clinical practice to treat hepatocellular carcinoma. YFJP demonstrated strong anticancer activity and prevented weight loss in H22 tumor-bearing mice. YFJP improved immune function and boosted CD8+ T cell and CD3+ T cell counts in H22 tumor-bearing mice’s spleen and peripheral blood.YFJP improved immune function and boosted CD8+ T cell and CD3+T cell counts in H22 tumor-bearing mice’s spleen and peripheral blood. YFJP also lowered the expression of PD-1, TIGIT, and TIM3 in CD8+ T cell and the production of IL-10, IL-4, IL-6, and IL-1*β* (87). Dahuang Fuzi Baijiang decoction (DFBD) is a combination of traditional prescriptions from the “Synopsis of Prescriptions of the Golden Chamber”. DFBD inhibits *CCL2* and maintains progenitor Tex in the obese microenvironment, slowing colorectal cancer development. This study provides convincing evidence that the traditional Chinese formula DFBD can inhibit tumor growth by modifying adaptive immunity, and it provides a solid basis for additional clinical testing (88). Sijunzi decoction was shown to suppress the expression of PD-1, PD-L1, and STAT3, as well as boost the killing impact of NK cell on subcutaneous tumors of rat colon cancer (89).

### 6.3 Study on CTLA-4 regulated by TCM

Advancements in sporoderm-breaking technologies have led to increased interest in active components from *Ganoderma Lucidum* and their diverse biological functions. The study found that spores of G. lucidum (ESG) suppressed 4T1 tumor development *in vivo* rather than *in vitro*. ESG might significantly increase the number of cytotoxic T cell (Tc) and the ratio of Tc to helper T cell (Th) in the peripheral blood of tumor-bearing mice; a similar increase in Tc cell was detected in tumor-infiltrating lymphocytes. ESG may act as a natural anticancer adjuvant by restoring depleted Tc cell levels, with significant therapeutic implications for breast cancer therapy (90).

Lycorine, an alkaloid extracted from plants of the Amaryllidaceae family, is touted as a potential anti-cancer drug due to its demonstrated capacity to inhibit growth (including induction of cell cycle arrest and inhibition of vasculogenic mimicry formation). Researchers have found that lycorine hydrochloride inhibits the viability of various renal cell carcinoma cell lines (91). Lycorine hydrochloride and anti-CTLA-4 synergistically decreased tumor weight, lung metastasis, and luciferin staining in tumor images. Importantly, the observed anti-tumor effects of this combination depended significantly on the suppression of regulatory T cell while upregulating effector T cell. Specifically, a decrease of 31.43% in regulatory T cell and an increase of 31.59% in effector T cell were observed in the combination group compared to the control group (92).

Cordycepin, an adenosine derivative composed of adenine and pentose, holds immense promise for treating inflammation and cancer. A study supported the idea that combining cordycepin with CTLA-4 blockade could modify the effector and exhaustion status of CD8+ T cell, thereby bolstering CD8+ T cell-mediated anti-tumor immunity in the TME. Furthermore, the combined treatment of cordycepin and an anti-CD47 antibody significantly curtailed tumor growth and extended the lifespan of tumor-bearing mice (93). Cheng et al. discovered that cordycepin combined with anti-TIGIT therapy led to a significant increase in the proportion of NK cell within the tumor immune microenvironment. In the combination therapy group, CD8+ T cell had lower exhaustion state scores and increased cytotoxicity, indicating a better immune response (94).

Yu et al. discovered that astragalus can regulate the frequency of Treg in the spleen of tumor-bearing mice and lower the expression levels of CTLA-4 mRNA and Foxp3 mRNA in tumor tissues using an animal model of Lewis lung cancer (95). Peng et al. discovered that Xiaoshuisan can decrease malignant ascites using a mouse peritoneal transplantation model. The expression of B7-1, B7-2, and CD28 increased in a dose-dependent manner, while CTLA-4 expression decreased (96). The Ling Jia anti-cancer compound extract has been shown to reduce the proportion of Treg in splenic lymphocytes. It down-regulates the expression of FOXP3 and CTLA-4, inhibits the release of cytokines such as TGF-*β* and IL-10, and restores immune function (97). In conclusion, TCM has the potential to boost the effectiveness of CTLA-4 inhibitors through multi-link immune regulation while also playing a larger role in tumor immune control.

### 6.4 TCM can prevent and cure immune checkpoint inhibitor-related side effects

Immune checkpoint medications that target PD-1/PD-L1 and CTLA-4 are being employed in a variety of cancers. However, as ICIs usage grows, so does the frequency of IRAEs. IRAEs may occur months after therapy, or even during the course of ICIs (98). Unlike typical cancer therapies, IRAEs is frequently treatable but occasionally deadly. IRAEs was reported in 70% of patients treated with PD-1/PD-L1 antibodies. IRAEs affects up to 90% of patients treated with anti-CTLA-4 antibodies (1). Common adverse reactions include: 1. Immune-related adverse effects, including rashes, pruritus, diarrhea, inflammatory bowel disease, and hepatitis; 2. Thyroiditis, diabetes, and other immune-enhancing responses; 3. Neurotoxicity, which might manifest as headaches, dizziness, lethargy, ataxia, and other symptoms; 4. Adverse pulmonary responses, including pneumonia or pulmonary fibrosis; 5. Cardiovascular side effects, including arrhythmia and myocarditis;6. Adverse effects on the endocrine system, such as hyperthyroidism and adrenal insufficiency (99).

Currently, IRAEs are thought to be associated with dysregulations in the body’s immune system, including loss of autoimmune tolerance or increased sensitivity to antigen recognition, leading to attacks on its own tissues. IRAEs can occur through various mechanisms, including synthesis of autoantibodies, infiltration of T cell, and mediation by inflammatory cytokines such as IL (100). PD-1 and PD-L1 inhibitors reduce Treg production, impair their function, and enhance cytokine production. CTLA-4 inhibitors induce several cellular changes, including T cell activation and proliferation, reduced Treg survival, and increased Th17 cell numbers. Additionally, CTLA-4 inhibitors can promote *in vivo* cross-reactivity and autoantibody synthesis between anti-tumor T cell and normal cell antigens. Human involvement can disrupt immunological tolerance mechanisms, leading to non-specific activation of immune cells that target normal tissues alongside tumor cells, causing excessive immune responses and inflammatory damage (101).

TCM is a significant therapy approach for reducing immunological inflammatory responses. Starting with clinical concerns, Li et al. performed a TCM syndrome differentiation study of IRAEs that emerged with PD-1 therapy. Shengmai decoction (*Ginseng Radix et Rhizoma Rubra, Ophiopogonis Radix, Schisandrae Chinensis Fructus*), in combination with Shashen Maidong decoction (*Glehniae radix, Ophiopogonis Radix, Polygonati Odorati Rhizoma, Glycyrrhizae Radix et Rhizoma, Lablab Semen Album, Mori folium, trichosanthis Radix*), can effectively alleviate immune-related tiredness symptoms and prevent the illness from worsening (102). Zhou et al. found that the combined treatment of Fufang Yifei Yin (*Pseudostellariae Radix, Angelicae Sinensis Radix, Phragmitis Rhizoma, Fagopyri Dibotryis Rhizoma, Salviae Miltiorrhizae Radix et Rhizoma, Glehniae Radix, Ophiopogonis Radix, Aurantii Fructus, Pinelliae Rhizoma*) and glucocorticoid had a good effect on reducing the grading of ICIs-related pneumonia. The combined drug group scored significantly lower than the glucocorticoid group alone (*P*<0.05), and tolerance was acceptable (103). Some academics think that invigorating the spleen and stomach with TCM or food, as well as pressing acupoints like *Zusanli* and *Neiguan*, helps alleviate IRAEs-related gastrointestinal symptoms such as nausea, vomiting, and diarrhea (104). Shen et al. discovered that external use of Zhiyang Pingfu Lotion (*Scutellariae Radix, Portulacae Herba, Dictamni Cortex, Sophorae Flavescentis Radix, Taraxaci Herba*) efficiently relieves skin irritation caused by IRAEs and improves quality of life. It might be connected to limiting the release of inflammatory factors (105). In prospective randomized research measuring renal function in 48 patients with ICIS-induced acute kidney damage, Qigui Yishen decoction (*Astragali Radix, Angelicae Sinensis Radix, Salviae Miltiorrhizae Radix et Rhizoma, Smilacis Glabrae Rhizoma, Atractylodis Macrocephalae Rhizoma, Hirudo, Paeoniae Radix Alba, Eucommiae Cortex, Codonopsis Radix, Rhei Radix et Rhizoma, Epimedii Folium, Lycii Fructus, Plantaginis Semen*) substantially lowered blood urea nitrogen, serum creatinine, and glomerular filtration rate when compared to corticosteroids alone (106). At the same time, the degree of acute renal damage from ICIs reduced. Immuno-associated cystitis has also been documented in cancer patients following 5 cycles of paclitaxel and tegafur with anti-PD-1 treatment. Two months after using Chailing decoction (*Bupleuri Radix, Pinelliae Rhizoma, Scutellariae Radix, Ginseng Radix et Rhizoma, Glycyrrhizae Radix et Rhizoma, Aatractylodis Macrocephalae Rhizoma, Polyporus, Poria, Alismatis Rhizoma, Cinnamomi Cortex*) orally, the patient restored to normal without relapse. Chailing decoction has been recommended as a safe and efficient therapy for immunological cystitis (107). In addition to herbal substances, acupuncture is used to treat limb numbness and exhaustion in individuals with immune-associated Guillain-Barré syndrome (108).

## 7 Discussion

While ICIs have shown significant therapeutic effects in cancer treatment, the development of medication resistance limits their effectiveness for many patients. Various approaches are being explored to enhance the efficacy of ICIs, including combining immunotherapy with conventional anticancer treatments such as chemotherapy, radiation, or targeted therapy. However, these combinations face challenges including primary and acquired resistance, variable effectiveness, and unpredictable toxicity. Finding medications that can enhance the sensitivity of tumor immunotherapy, reduce toxicity, and improve overall immune function remains a critical goal in medicine. Addressing this challenge requires combination therapies that leverage synergistic effects at a complex molecular level.

TCM in China has made significant strides in cancer research, showing promising potential. Recent studies indicate that TCM can enhance sensitivity to cancer immunotherapy by directly targeting the PD-1/PD-L1 axis, modulating T cell activity, suppressing immunosuppressive cells, improving the TME, and regulating gut flora. TCM offers diverse treatment options tailored to various tumor stages, categorized by syndrome differentiation.

TCM not only enhances the therapeutic effectiveness of ICIs but also shows potential in preventing and treating IRAEs, a novel concept. TCM, with its diverse components, targets, and administration routes, may offer synergistic benefits and mitigate IRAEs. However, several challenges remain in current studies. Firstly, discrepancies exist between basic research and clinical outcomes. Secondly, the suitability of existing clinical efficacy evaluation standards for assessing TCM combined with ICIs needs to be validated. Next, the relationship between the content of TCM components and pharmacological activity needs to be further explored. Lastly, the mechanisms underlying the combination of TCM and ICIs require further investigation. Therefore, grounded in TCM principles, there is a critical need to explore the interaction between TCM and immunotherapy comprehensively, expand our perspectives, and identify additional agents that can counteract immune evasion. Additionally, it is essential to develop medical evaluation criteria that accommodate TCM combined with ICIs and establish the use of evidence-based standardized treatments.

## References

[1] Topalian SL Forde PM Emens LA Yarchoan M Smith KN Pardoll DM. Neoadjuvant immune checkpoint blockade: A window of opportunity to advance cancer immunotherapy. Cancer Cell. (2023) 41:1551–66. doi: 10.1016/j.ccell.2023.07.011 PMC10548441 37595586

[2] Ren Z Yang K Zhu L Yin D Zhou Y. Regulatory T cells as crucial trigger and potential target for hyperprogressive disease subsequent to PD-1/PD-L1 blockade for cancer treatment. Int Immunopharmacol. (2024) 132:111934. doi: 10.1016/j.intimp.2024.111934 38574701

[3] Sharma N Fan X Atolagbe OT Ge Z Dao KN Sharma P. ICOS costimulation in combination with CTLA-4 blockade remodels tumor-associated macrophages toward an antitumor phenotype. J Exp Med. (2024) 221:e20231263. doi: 10.1084/jem.20231263 38517331 PMC10959121

[4] Borelli B Antoniotti C Carullo M Germani MM Conca V Masi G. Immune-checkpoint inhibitors (ICIs) in metastatic colorectal cancer (mCRC) patients beyond microsatellite instability. Cancers (Basel). (2022) 14:4974. doi: 10.3390/cancers14204974 36291761 PMC9599678

[5] Diaz LA Jr Shiu KK Kim TW Jensen BV Jensen LH Punt C. Pembrolizumab versus chemotherapy for microsatellite instability-high or mismatch repair-deficient metastatic colorectal cancer (KEYNOTE-177): final analysis of a randomised, open-label, phase 3 study. Lancet Oncol. (2022) 3:659–70. doi: 10.1016/S1470-2045(22)00197-8 PMC9533375 35427471

[6] Rizvi N Ademuyiwa FO Cao ZA Chen HX Ferris RL Goldberg SB. Society for Immunotherapy of Cancer (SITC) consensus definitions for resistance to combinations of immune checkpoint inhibitors with chemotherapy. J Immunother Cancer. (2023) 11:e005920. doi: 10.1136/jitc-2022-005920 36918220 PMC10016262

[7] Peng J Yin X Yun W Meng X Huang Z. Radiotherapy-induced tumor physical microenvironment remodeling to overcome immunotherapy resistance. Cancer Lett. (2023) 559:216108. doi: 10.1016/j.canlet.2023.216108 36863506

[8] Abulizi A Yan G Xu Q Muhetaer R Wu S Abudukelimu K. Cardiovascular adverse events and immune-related adverse events associated with PD-1/PD-L1 inhibitors for head and neck squamous cell carcinoma (HNSCC). Sci Rep. (2024) 14:25919. doi: 10.1038/s41598-024-75099-5 39472591 PMC11522629

[9] Wei SC Duffy CR Allison JP. Fundamental mechanisms of immune checkpoint blockade therapy. Cancer Discovery. (2018) 8:1069–86. doi: 10.1158/2159-8290.CD-18-0367 30115704

[10] Pauken KE Torchia JA Chaudhri A Sharpe AH Freeman GJ. Emerging concepts in PD-1 checkpoint biology. Semin Immunol. (2021) 52:101480. doi: 10.1016/j.smim.2021.101480 34006473 PMC8545711

[11] Meci A Goyal N Slonimsky G. Mechanisms of resistance and therapeutic perspectives in immunotherapy for advanced head and neck cancers. Cancers (Basel). (2024) 16:703. doi: 10.3390/cancers16040703 38398094 PMC10887076

[12] Xu Y Liu Y Ge Y Li H Zhang Y Wang L. Drug resistance mechanism and reversal strategy in lung cancer immunotherapy. Front Pharmacol. (2023) 14:1230824. doi: 10.3389/fphar.2023.1230824 37795038 PMC10546211

[13] O’Melia MJ Manspeaker MP Thomas SN. Tumor-draining lymph nodes are survival niches that support T cell priming against lymphatic transported tumor antigen and effects of immune checkpoint blockade in TNBC. Cancer Immunol Immunother. (2021) 70:2179–95. doi: 10.1007/s00262-020-02792-5 PMC8286278 33459842

[14] De Lorenzo S Tovoli F Trevisani F. Mechanisms of primary and acquired resistance to immune checkpoint inhibitors in patients with hepatocellular carcinoma. Cancers (Basel). (2022) 14:4616. doi: 10.3390/cancers14194616 36230538 PMC9564277

[15] Gong J Robertson MD Kim E Fakih M Schrock AB Tam KW. Efficacy of PD-1 blockade in refractory microsatellite-stable colorectal cancer with high tumor mutation burden. Clin Colorectal Cancer. (2019) 18:307–9. doi: 10.1016/j.clcc.2019.08.001 31563511

[16] Sidaway P. MSI-H/dMMR mCRC: ICIs in the first line? Nat Rev Clin Oncol. (2021) 18:748. doi: 10.1038/s41571-021-00576-z 34697487

[17] Williams CJM Peddle AM Kasi PM Seligmann JF Roxburgh CS Middleton GW. Neoadjuvant immunotherapy for dMMR and pMMR colorectal cancers: therapeutic strategies and putative biomarkers of response. Nat Rev Clin Oncol. (2024). doi: 10.1038/s41571-024-00943-6 39317818

[18] Sun Q Wei X Wang Z Zhu Y Zhao W Dong Y. Primary and Acquired Resistance against Immune Check Inhibitors in Non-Small Cell Lung Cancer. Cancers (Basel). (2022) 14:3294. doi: 10.3390/cancers14143294 35884355 PMC9316464

[19] Lu D Zhang H Zhang Y Zhao G Anwar Khan F Chen Y. Secreted mbovP0145 promotes IL-8 expression through its interactive β-actin and MAPK activation and contributes to neutrophil migration. Pathogens. (2021) 10:1628. doi: 10.3390/pathogens10121628 34959583 PMC8707762

[20] Álvarez-Garcia V Tawil Y Wise HM Leslie NR. Mechanisms of PTEN loss in cancer: It’s all about diversity. Semin Cancer Biol. (2019) 59:66–79. doi: 10.1016/j.semcancer.2019.02.001 30738865

[21] Sanchez-Pupo RE Finch GA Johnston DE Craig H Abdo R Barr K. Global pannexin 1 deletion increases tumor-infiltrating lymphocytes in the BRAF/Pten mouse melanoma model. Mol Oncol. (2024) 18:969–87. doi: 10.1002/1878-0261.13596 PMC10994229 38327091

[22] Bulati M Miceli V Gallo A Amico G Carcione C Pampalone M. The immunomodulatory properties of the human amnion-derived mesenchymal stromal/stem cells are induced by INF-γ Produced by activated lymphomonocytes and are mediated by cell-to-cell contact and soluble factors. Front Immunol. (2020) 11:54. doi: 10.3389/fimmu.2020.00054 32117234 PMC7028706

[23] Wang K Wang J Liu T Yu W Dong N Zhang C. Morphine-3-glucuronide upregulates PD-L1 expression via TLR4 and promotes the immune escape of non-small cell lung cancer. Cancer Biol Med. (2021) 18:155–71. doi: 10.20892/j.issn.2095-3941.2020.0442 PMC7877184 33628591

[24] Chehrazi-Raffle A Dorff TB Pal SK. Wnt/β-catenin signaling and immunotherapy resistance: lessons for the treatment of urothelial carcinoma. Cancers (Basel). (2021) 13:889. doi: 10.3390/cancers13040889 33672668 PMC7924395

[25] Mortezaee K. WNT/β-catenin regulatory roles on PD-(L)1 and immunotherapy responses. Clin Exp Med. (2024) 24:15. doi: 10.1007/s10238-023-01274-z 38280119 PMC10822012

[26] Dong M Wang HY Zhao XX Chen JN Zhang YW Huang Y. Expression and prognostic roles of PIK3CA, JAK2, PD-L1, and PD-L2 in Epstein-Barr virus-associated gastric carcinoma. Hum Pathol. (2016) 3:25–34. doi: 10.1016/j.humpath.2016.02.007 26980034

[27] Schoenfeld AJ Hellmann MD. Acquired resistance to immune checkpoint inhibitors. Cancer Cell. (2020) 37:443–55. doi: 10.1016/j.ccell.2020.03.017 PMC7182070 32289269

[28] Meyer M Mahr A Brewer J Daniel V Dell’Aringa J Goldstone T. A call to adapt the regulation of HLA testing for T cell receptor-based therapeutics. Nat Rev Drug Discovery. (2024) 23:1–2. doi: 10.1038/d41573-023-00189-4 38030734

[29] Mukherjee S Ghosh P Ghosh S Sengupta A Sarkar S Chatterjee R. Administration of rIL-33 Restores Altered mDC/pDC Ratio, MDSC Frequency, and Th-17/Treg Ratio during Experimental Cerebral Malaria. Pathogens. (2024) 13:877. doi: 10.3390/pathogens13100877 39452748 PMC11509898

[30] Zhu Y Chen P Hu B Zhong S Yan K Wu Y. MDSC-targeting gold nanoparticles enhance PD-1 tumor immunotherapy by inhibiting NLRP3 inflammasomes. Biomaterials. (2024) 307:122533. doi: 10.1016/j.biomaterials.2024.122533 38493671

[31] Wang JC Chen CC Manvar K Sun LS Joseph G Wong C. *In Vitro* Drug Studies of Inhibiting MDSC to Improve Efficacy of ICI (immune check point inhibitor) Therapy in Ph(-) myeloproliferative Neoplasm. Blood. (2022) 140:12187–8. doi: 10.1182/blood-2022-158403

[32] Han SC Kang JI Choi YK Boo HJ Yoon WJ Kang HK. Intermittent fasting modulates immune response by generating tregs via TGF-β Dependent mechanisms in obese mice with allergic contact dermatitis. Biomol Ther (Seoul). (2024) 32:136–45. doi: 10.4062/biomolther.2023.053 PMC10762271 37424516

[33] Dąbrowska A Grubba M Balihodzic A Szot O Sobocki BK Perdyan A. The role of regulatory T cells in cancer treatment resistance. Int J Mol Sci. (2023) 24:14114. doi: 10.3390/ijms241814114 37762416 PMC10531820

[34] John P Pulanco MC Galbo PM Jr Wei Y Ohaegbulam KC Zheng D. The immune checkpoint B7x expands tumor-infiltrating Tregs and promotes resistance to anti-CTLA-4 therapy. Nat Commun. (2022) 3:2506. doi: 10.1038/s41467-022-30143-8 PMC9076640 35523809

[35] Zhang X Wang J Liu N Wu W Li H Lu W. Umbilical cord blood-derived M1 macrophage exosomes loaded with cisplatin target ovarian cancer *in vivo* and reverse cisplatin resistance. Mol Pharm. (2023) 20:5440–53. doi: 10.1021/acs.molpharmaceut.3c00132 37819754

[36] Lin H Fu L Zhou X Yu A Chen Y Liao W. LRP1 induces anti-PD-1 resistance by modulating the DLL4-NOTCH2-CCL2 axis and redirecting M2-like macrophage polarisation in bladder cancer. Cancer Lett. (2024) 216807. doi: 10.1016/j.canlet.2024.216807 38462037

[37] Sandoval-Hernández MA Fierro NA Veytia-Bucheli JI Alvarado-Velázquez DA Alemán-Navarro E Melchy-Pérez E. Differential impact of CD43 and CD28 on T-cell differentiation depending on the order of engagement with the TCR. Int J Mol Sci. (2024) 25:3135. doi: 10.3390/ijms25063135 38542109 PMC10970108

[38] Piao W Li L Saxena V Iyyathurai J Lakhan R Zhang Y. PD-L1 signaling selectively regulates T cell lymphatic transendothelial migration. Nat Commun. (2022) 13:2176. doi: 10.1038/s41467-022-29930-0 35449134 PMC9023578

[39] Lv ZB Zhang JJ Xiang C. GDF10 and IDO1 as a thyroid cancer prognostic biomarker associated with immune infiltration. Heliyon. (2024) 10:e27651. doi: 10.1016/j.heliyon.2024.e27651 38509876 PMC10950683

[40] Ouyang Y Zhong W Xu P Wang B Zhang L Yang M. Tumor-associated neutrophils suppress CD8+ T cell immunity in urothelial bladder carcinoma through the COX-2/PGE2/IDO1 Axis. Br J Cancer. (2024) 130:880–91. doi: 10.1038/s41416-023-02552-z PMC10912642 38233491

[41] Astrid K Thomas G Sven W Nolte E Kappler M Bache M. High coexpression of CCL2 and CX3CL1 is gender-specifically associated with good prognosis in soft tissue sarcoma patients. Int J Cancer. (2014) 135:2096–106. doi: 10.1002/ijc.28867 24676787

[42] Zhu Y Banerjee A Xie P Ivanov AA Uddin A Jiao Q. Pharmacological suppression of the OTUD4/CD73 proteolytic axis revives antitumor immunity against immune-suppressive breast cancers. J Clin Invest. (2024) 134(10):e176390. doi: 10.1172/JCI176390 38530357 PMC11093616

[43] Giraulo C Turiello R Orlando L Leonardelli S Landsberg J Belvedere R. The CD73 is induced by TGF-β1 triggered by nutrient deprivation and highly expressed in dedifferentiated human melanoma. BioMed Pharmacother. (2023) 165:115225. doi: 10.1016/j.biopha.2023.115225 37517292

[44] Leikeim L Li H An L Sticht C Krämer BK Yard B. Adenosine signalling in T-cell activation favours development of IL-17 positive cells with suppressive properties. Immunology. (2023) 169:42–56. doi: 10.1111/imm.v169.1 36373432

[45] Khosravi GR Mostafavi S Bastan S Ebrahimi N Gharibvand RS Eskandari N. Immunologic tumor microenvironment modulators for turning cold tumors hot. Cancer Commun (Lond). (2024) 44(5):521–53. doi: 10.1002/cac2.12539 PMC11110955 38551889

[46] Liu B Liu Z Jiang T Gu X Yin X Cai Z. Univariable and multivariable Mendelian randomization study identified the key role of gut microbiota in immunotherapeutic toxicity. Eur J Med Res. (2024) 29:161. doi: 10.1186/s40001-024-01741-7 38475836 PMC10929167

[47] Rosenbaum SR Wilski NA Aplin AE. Fueling the fire: inflammatory forms of cell death and implications for cancer immunotherapy. Cancer Discovery. (2021) 11:266–81. doi: 10.1158/2159-8290.CD-20-0805 PMC7858229 33451983

[48] Zhang C Fu F Zhu X Ni X Yue S Wu H. Intermittent fasting and fasting-mimicking diet: promising strategies in cancer management. Curr medicinal Chem. (2024). doi: 10.2174/0109298673332052241008060857 39449338

[49] Yang Q Meng D Zhang Q. Advances in research on the anti-tumor mechanism of Astragalus polysaccharides. Front Oncol. (2024) 14:1334915. doi: 10.3389/fonc.2024.1334915 38515577 PMC10955345

[50] Wen Y Li Y Li BB Liu P Qiu M Li Z. Pyroptosis induced by natural products and their derivatives for cancer therapy. Biomateri Sci. (2024) 12(22):5656–79. doi: 10.1039/D4BM01023J 39429101

[51] Zhang R Shi P Chou Y Liu W Zhang C. The effect of traditional Chinese medicine on psychological conditions among elderly patients with cancer: a scoping review. Psychogeriatrics. (2024) 24:1389–401. doi: 10.1111/psyg.13182 PMC12997152 39209532

[52] Wang W Fan J Zhang C Huang Y Chen Y Fu S. Targeted modulation of gut and intra-tumor microbiota to improve the quality of immune checkpoint inhibitor responses. Microbiol Res. (2024) 282:127668. doi: 10.1016/j.micres.2024.127668 38430889

[53] Wang SY Wu LGJ Chen B Deng WL. Current research status of strengthening body resistance chinese herbs regulating immunosuppressive tumor microenvironment. Modernization Traditional Chin Med Materia Medica-World Sci Technol. (2022) 24:3862–8. doi: 10.11842/wst.20211008005

[54] Huang J Liu D Wang Y Liu L Li J Yuan J. Ginseng polysaccharides alter the gut microbiota and kynurenine/tryptophan ratio, potentiating the antitumour effect of antiprogrammed cell death 1/programmed cell death ligand 1 (anti-PD-1/PD-L1) immunotherapy. Gut. (2022) 71:734–45. doi: 10.1136/gutjnl-2020-321031 PMC8921579 34006584

[55] Han X Wei Q Lv Y Cai X Cao M Cao P. Ginseng-derived nanoparticles potentiate immune checkpoint antibody efficacy by reprogramming the cold tumor microenvironment. Mol Ther. (2022) 30:327–40. doi: 10.1016/j.ymthe.2021.08.028 PMC8753455 34450250

[56] Li J-j Yang J-y Sun F-f Wang L. Regulatory effects of astragalus polysaccharides on PD 1/PD Ll expression in Lewis tumor- bearing mouse model. Tianjin Pharm. (2022) 34. 9-13 + 37.

[57] Koch M Rennert J Schulz C. Pancerebellitis under immunotherapy with pembrolizumab. Dtsch Arztebl Int. (2022) 119:820. doi: 10.3238/arztebl.m2022.0228 PMC9906027 36747446

[58] Yao N Ma Q Yi W Zhu Y Liu Y Gao X. Artesunate attenuates the tumorigenesis of choroidal melanoma via inhibiting EFNA3 through Stat3/Akt signaling pathway. J Cancer Res Clin Oncol. (2024) 150:202. doi: 10.1007/s00432-024-05711-8 38630320 PMC11024049

[59] Liang C Ma L Chen Y Li J Wang B Ma C. Artesunate alleviates kidney fibrosis in type 1 diabetes with periodontitis rats via promoting autophagy and suppression of inflammation. ACS Omega. (2024) 9:16358–73. doi: 10.1021/acsomega.4c00020 PMC11007779 38617690

[60] Nguyen CDK Chun-Ling YI. YAP/TAZ signaling and resistance to cancer therapy. Trends Cancer. (2019) 5:283–96. doi: 10.1016/j.trecan.2019.02.010 PMC6557283 31174841

[61] Lakra DS Bharathiraja P Dhanalakshmi T Prasad NR. Andrographolide reverts multidrug resistance in KBChR 8-5 cells through AKT signaling pathway. Cell Biochem Funct. (2024) 2:e3948. doi: 10.1002/cbf.v42.2 38379216

[62] Liu W Fan T Li M Zhang G Guo W Yang X. Andrographolide potentiates PD - 1 blockade immunotherapy by inhibiting COX2-mediated PGE2 release. Int Immunopharmacol. (2020) 81:106206. doi: 10.1016/j.intimp.2020.106206 32018066

[63] Li W Xu D Li B Cao N Guo S Jiang Q. The polysaccharide of Atractylodes macrocephala koidz (PAMK) alleviates cyclophosphamide-mediated immunosuppression in geese, possibly through novel_mir2 targeting of CTLA4 to upregulate the TCR-NFAT pathway. RSC Adv. (2018) 8:26837–48. doi: 10.1039/C8RA00368H PMC9083368 35541089

[64] Han YC Chen YL Fan XQ Shang YW Chen X Wang G. Polysaccharide of Atractylodis Macrocephalae Rhizoma inhibits expression ofimmune checkpoint PD-L1 by targeting miR-34a in esophageal carcinoma cells. China J Chin Materia Med. (2022) 47:1658–65. doi: 10.19540/j.cnki.cjcmm.20211203.701 35347965

[65] Cen Q Chen J Guo J Chen M Wang H Wu S. CLPs-miR-103a-2-5p inhibits proliferation and promotes cell apoptosis in AML cells by targeting LILRB3 and Nrf2/HO-1 axis, regulating CD8 + T cell response. J Transl Med. (2024) 22:278. doi: 10.1186/s12967-024-05070-5 38486250 PMC10938737

[66] Wang T Chen Z Chen H Yu X Wang L Liu X. Brusatol inhibits the growth of renal cell carcinoma by regulating the PTEN/PI3K/AKT pathway. J Ethnopharmacol. (2022) 288:115020. doi: 10.1016/j.jep.2022.115020 35066068

[67] Oh ET Kim CW Kim HG Lee JS Park HJ. Brusatol-Mediated Inhibition of c-Myc Increases HIF-1αDegradation and Causes Cell Death in Colorectal Cancer under Hypoxia. Theranostics. (2017) 7:3415–31. doi: 10.7150/thno.20861 PMC5596433 28912885

[68] Liu S Han Z Trivett AL Lin H Hannifin S Yang D. Cryptotanshinone has curative dual anti-proliferative and immunotherapeutic effects on mouse Lewis lung carcinoma. Cancer Immunol Immunother. (2019) 68:1059–71. doi: 10.1007/s00262-019-02326-8 PMC6584267 30972427

[69] Chen Q Hong Y Weng S Guo P Li B Zhang Y. Traditional chinese medicine pien-tze-huang inhibits colorectal cancer growth and immune evasion by reducing β - catenin transcriptional activity and PD-L1 expression. Front Pharmacol. (2022) 13:828440. doi: 10.3389/fphar.2022.828440 35185580 PMC8850789

[70] Jing L Lin J Yang Y Tao L Li Y Liu Z. Quercetin inhibiting the PD-1/PD-L1 interaction for immune-enhancing cancer chemopreventive agent. Phytother Res. (2021) 35:6441–51. doi: 10.1002/ptr.v35.11 34560814

[71] Almohammad Aljabr B Zihlif M Abu-Dahab R Zalloum H. Effect of quercetin on doxorubicin cytotoxicity in sensitive and resistant human MCF7 breast cancer cell lines. BioMed Rep. (2024) 20:58. doi: 10.3892/br.2024.1745 38414625 PMC10895388

[72] Hayakawa T Yaguchi T Kawakami Y. Enhanced anti-tumor effects of the PD-1 blockade combined with a highly absorptive form of curcumin targeting STAT3. Cancer Sci. (2020) 111:4326–35. doi: 10.1111/cas.v111.12 PMC7734012 33006786

[73] Dai C Zhou X Wang L Tan R Wang W Yang B. Rocaglamide prolonged allograft survival by inhibiting differentiation of th1/th17 cells in cardiac transplantation. Oxid Med Cell longevity. (2022) 2022:2048095. doi: 10.1155/2022/2048095 PMC8787457 35087613

[74] Feng Y An Q Zhao Z Wu M Yang C Liang W. Beta-elemene: A phytochemical with promise as a drug candidate for tumor therapy and adjuvant tumor therapy. BioMed Pharmacother. (2024) 172:116266. doi: 10.1016/j.biopha.2024.116266 38350368

[75] Feng YX Li CC Liu SWYan F Teng Y Li XY. β-Elemene Restrains PTEN mRNA Degradation to Restrain the Growth of Lung Cancer Cells via METTL3-Mediated N6 Methyladenosine Modification. J Oncol. (2022) 2022:3472745. doi: 10.1155/2022/3472745 35069732 PMC8769858

[76] Wang H-Y Ma Y-Y. β-Elemene alleviates cisplatin resistance in oral squamous cell carcinoma cell via inhibiting JAK2/STAT3 pathway *in vitro* and *in vivo* . Cancer Cell Int. (2022) 22(1):244. doi: 10.1186/s12935-022-02650-7 35909161 PMC9341059

[77] Zou WW Xu SP. Galangin inhibits the cell progression and induces cell apoptosis through activating PTEN and Caspase-3 pathways in retinoblastoma. BioMed Pharmacother. (2018) 97:851–63. doi: 10.1016/j.biopha.2017.09.144 29136761

[78] Zhong Y Li MY Han L Tai Y Cao S Li J. Galangin inhibits programmed cell death-ligand 1 expression by suppressing STAT3 and MYC and enhances T cell tumor-killing activity. Phytomedicine. (2023) 116:154877. doi: 10.1016/j.phymed.2023.154877 37267692

[79] Yu P Wei H Li K Zhu S Li J Chen C. The traditional chinese medicine monomer Ailanthone improves the therapeutic efficacy of anti-PD-L1 in melanoma cells by targeting c-Jun. J Exp Clin Cancer Res. (2022) 1:346. doi: 10.1186/s13046-022-02559-z PMC9753288 36522774

[80] Yan X Yao C Fang C Han M Gong C Hu D. Rocaglamide promotes the infiltration and antitumor immunity of NK cells by activating cGAS-STING signaling in non-small cell lung cancer. Int J Biol Sci. (2022) 18:585–98. doi: 10.7150/ijbs.65019 PMC8741839 35002511

[81] Tang Q Xie J Jiang X Wang MM Guo W Liang C. Exploring the mechanism and key active components of Gegen Qinlian decoction combined with XELOX in the treatment of ulcerative colitis-associated colorectal cancer based on network pharmacology and multi-omics analysis. Arabian J Chem. (2024) 17:105625. doi: 10.1016/j.arabjc.2024.105625

[82] Ma Y Qi Y Zhou Z Yan Y Chang J Zhu X. Shenqi Fuzheng injection modulates tumor fatty acid metabolism to downregulate MDSCs infiltration, enhancing PD-L1 antibody inhibition of intracranial growth in Melanoma. Phytomedicine. (2024) 122:155171. doi: 10.1016/j.phymed.2023.155171 37925891

[83] Pan J Yang H Zhu L Lou Y Jin B. Qingfei Jiedu decoction inhibits PD-L1 expression in lung adenocarcinoma based on network pharmacology analysis, molecular docking and experimental verification. Front Pharmacol. (2022) 13:897966. doi: 10.3389/fphar.2022.897966 36091822 PMC9454399

[84] Li X-J Zhu W-w Miu Z-y Zhou YM. Mechanism of jianpi zishen xiehuo recipe in improving primary immune thrombocytopenia by LMP2AJAK/STAT signaling pathway. Pharmacol Clinics Chin Materia Med., 1–12. doi: 10.13412/j.cnki.zyyl.20230321.001

[85] Yang X-j. Clinical study on the effect of the TCM strengthening the spleen and complement the kidney on the serum complement and complement regulator protein CD55 and CD59 levels in the systemic lupus erythemators patients. Anhui Province: Anhui University of Chinese Medicine (2016).

[86] Xu R Wu J Zhang X Zou X Li C Wang H. Modified Bu-zhong-yi-qi decoction synergies with 5 fluorouracile to inhibits gastric cancer progress via PD-1/PD-L1-dependent T cell immunization. Pharmacol Res. (2020) 152:104623. doi: 10.1016/j.phrs.2019.104623 31899315

[87] Xie Y Yan F Wang X Yu L Yan H Pu Q. Mechanisms and network pharmacological analysis of Yangyin Fuzheng Jiedu prescription in the treatment of hepatocellular carcinoma. Cancer Med. (2023) 12:3237–59. doi: 10.1002/cam4.v12.3 PMC9939140 36043445

[88] Xu Y Wang H Wang T Chen C Sun R Yao W. Dahuang Fuzi Baijiang decoction restricts progenitor to terminally exhausted T cell differentiation in colorectal cancer. Cancer Sci. (2022) 113:1739–51. doi: 10.1111/cas.v113.5 PMC9128181 35238098

[89] Zhu Y-y Song Y-l Shi X-l. Effect of Sijunzi Decoction on NK cells and colon cancer based on expression of PD-1/PD-L1. Chin J Immunol. (2021) 37:295–300 + 306. doi: 10.3969/j.issn.1000-484X.2021.03.007

[90] Su J Su L Li D Shuai O Zhang Y Liang H. Antitumor activity of extract from the sporoderm-breaking spore of ganoderma lucidum: restoration on exhausted cytotoxic t cell with gut microbiota remodeling. Front Immunol. (2018) 9:1765. doi: 10.3389/fimmu.2018.01765 30108589 PMC6079217

[91] Jiang F Zheng Q Zhao Q Qi Z Wu D Li W. Magnetic propelled hydrogel microrobots for actively enhancing the efficiency of lycorine hydrochloride to suppress colorectal cancer. Front Bioeng Biotechnol. (2024) 12:1361617. doi: 10.3389/fbioe.2024.1361617 38449675 PMC10915283

[92] Li X Xu P Wang C Xu N Xu A Xu Y. Synergistic effects of the immune checkpoint inhibitor CTLA-4 combined with the growth inhibitor lycorine in a mouse model of renal cell carcinoma. Oncotarget. (2017) 8:21177–86. doi: 10.18632/oncotarget.15505 PMC5400575 28416753

[93] Chen L Zheng X Huang H Feng C Wu S Chen R. Cordycepin synergizes with CTLA-4 blockade to remodel the tumor microenvironment for enhanced cancer immunotherapy. Int Immunopharmacol. (2023) 124:110786. doi: 10.1016/j.intimp.2023.110786 37611443

[94] Chen R Feng C Chen L Zheng X Fang W Wu S. Single-cell RNA sequencing indicates cordycepin remodels the tumor immune microenvironment to enhance TIGIT blockade’s anti-tumor effect in colon cancer. Int Immunopharmacol. (2024) 126:111268. doi: 10.1016/j.intimp.2023.111268 37992442

[95] Yu MW Sun GZ Qi X Li DR Zhang PT Wu J. Expressions of CD4 + and CD25 + of Tumor-bearing mice and Its Regulatory Molecules Intervened by Caesalpinina Sappan and Caesalpinina Sappan matched *Radix Astragali* . Chin J Basic Med Trad Chin Med. (2010) 16:384–6. doi: 10.19945/j.cnki.issn.1006-3250.2010.05.014

[96] Peng YH Peng W. Effect of Xiaoshui powder on the B7/CD28/cytotoxic T lymphocyte–associated antigen 4 co-stimulation pathway and ascites microenvironment in a xenograft tumor mouse model of human colorectal cancer. HUNAN J Trad C hin Med. (2023) 39:176–81. doi: 10.16808/j.cnki.issn1003-7705.2023.02.039

[97] Pan YL Zhang WC Chen J Zhou C Ma YX Li CY. The anti-tumor immune response mechanism of Lingjia anti-tumor medicine in tumor-bearing mice. Modern Oncol. (2016) 24:1699–704. doi: 10.3969/j.issn.1672-4992.2016.11.007

[98] Cariou PL Pobel C Michot JM Danlos FX Besse B Carbonnel F. Impact of immunosuppressive agents on the management of immune-related adverse events of immune checkpoint blockers. Eur J Cancer. (2024) 204:114065. doi: 10.1016/j.ejca.2024.114065 38643707

[99] Hong B Du B Chen R Zheng C Ni R Liu M. Comparison of immune checkpoint inhibitors related to pulmonary adverse events: a retrospective analysis of clinical studies and network meta-analysis. BMC Med. (2024) 22:75. doi: 10.1186/s12916-024-03285-3 38373990 PMC10875746

[100] Esfahani K Elkrief A Calabrese C Lapointe R Hudson M Routy B. Moving towards personalized treatments of immune-related adverse events. Nat Rev Clin Oncol. (2020) 17(8):504–15. doi: 10.1038/s41571-020-0352-8 32246128

[101] Galle P Finn RS Mitchell CR Ndirangu K Ramji Z Redhead GS. Treatment-emergent antidrug antibodies related to PD-1, PD-L1, or CTLA-4 inhibitors across tumor types: a systematic review. J Immunother Cancer. (2024) 12:e008266. doi: 10.1136/jitc-2023-008266 38238030 PMC10806538

[102] Li MH Chai SZ Dong ZY Ju LX. Clinical analysis of PD-1 monoclonal antibody supplemented with traditional Chinese medicine in the treatment of non-small cell lung cancer. SH J TCM. (2021) 55:28–30, 33. doi: 10.16305/j.1007-1334.2021.2012144

[103] Zhou Y Wei Y Chen JB Yu ZH Chai KJ. Clinical observation of Yifeiyin in treating pneumonia associated with immune checkpoint inhibitors. Zhejiang J Tradit Chin Med. (2021) 56:561–3. doi: 10.13633/j.cnki.zjtcm.2021.08.009

[104] Li YX Li S. Application of integrated Chinese and Western medicine nursing in patients treated with PD-1 inhibitor. Electronic J Of Pract Clin Nurs Sci. (2019) 4:41 + 60.

[105] Shen W Peng YM Zhen SY Zhang JY Zhang X Zhng YC. Two cases and literature review of immunorelated skin adverse reactions treated with Zhiprurigo Pingfu. J Trad Chin Med. (2020) 61:733–6. doi: 10.13288/j.11-2166/r.2020.08.021

[106] Wu LY Mi SC Xu ZJ He W Su YT Zhou YN. Clinical study of Qigui Yishen Decoction in treating acute kidney injury of tumor patients after application of PD-1/PD-L1 inhibitor. China MODERN DOCTOR. (2021) 59:124–8.

[107] Wang Z Zhu L Huang Y Peng L. Successful treatment of immune-related cystitis by Chai-Ling-Tang (Sairei-To) in a gastric carcinoma patient: Case report and literature review. Explore (NY). (2023) 19:458–62. doi: 10.1016/j.explore.2022.04.002 35469747

[108] Li J Xu D Liu Y Cao Y He J Liao M. Acupuncture treatment of guillain-barré syndrom e after using immune checkpoint inhibitors: A case report. Front Neurol. (2022) 13:908282. doi: 10.3389/fneur.2022.908282 35720101 PMC9201402

